# The role of Piezo1 in immune cells and pregnancy

**DOI:** 10.3389/fimmu.2026.1748854

**Published:** 2026-02-18

**Authors:** Yiming Fei, Yilin Liu, Lijuan Zhang

**Affiliations:** Department of Obstetrics and Gynecology, Shengjing Hospital of China Medical University, Shenyang, Liaoning, China

**Keywords:** adaptive immune responses, immune cells, innate immune system, Piezo1, pregnancy

## Abstract

In innate immunity, Piezo1 influences macrophage polarization, neutrophil migration, inflammation and repair. In adaptive immunity, it modulates T cell mechanosensation, immune synapse formation and B cell antigen presentation. Expressed in endometrium, placental vasculature, placental trophoblast and myometrium, Piezo1 regulates Ca²^+^ signaling during implantation, NO-dependent placental vasodilation, uterine contractions and inflammation, impacting pregnancy maintenance and labor onset. This review systematically summarizes the pivotal role of the mechanosensitive ion channel Piezo1 in immunoregulation and pregnancy. Firstly, it outlines the molecular structure of Piezo1 alongside its activation and inactivation mechanisms. Secondly, it focuses on Piezo1’s functions within innate and adaptive immune cells (such as macrophages and T cells) and its regulation of immune responses. Subsequently, it delves into Piezo1’s pivotal role across multiple stages of pregnancy, including its influence on uteroplacental vascular remodeling, trophoblast cell fusion, labor initiation, and fetal development. Particular attention is given to its potential bridging function linking immune homeostasis with successful pregnancy. Finally, it outlines the therapeutic potential of targeting Piezo1 in related pregnancy disorders and future research directions. This paper aims to provide insights into the integrated function of mechanobiology within reproductive immunity.

## 1 Introduction

The immune system undergoes significant changes during pregnancy, which are essential for the mother’s and the fetus’s well-being due to the intricate interplay between the two systems (1). Evidence from animal research suggests that a complex web of interactions between different immune effector cells and molecules is essential for the development of maternal immunological tolerance throughout pregnancy. Different T cell subtypes play distinct roles during pregnancy (2). For example, Th1 cells dominate during the embryo implantation period and can induce trophoblast cell invasion. As placental tissue develops, the anti-inflammatory function of Th2 cells supports fetal development and maintains the normal course of pregnancy (3). In the uterine microenvironment, innate immune cells including macrophages and natural killer (NK) cells also modify their phenotype and function in reaction to uterine environment changes (4). Additionally, the immune system undergoes changes as labor is initiated (5), and in-depth studies on fetal development mechanisms show that immune cells not only protect the fetus from infections but also participate in fetal growth and development.

As our understanding of how cells respond to mechanical stimuli has expanded, Piezo ion channels have been identified as mechanosensitive ion channel. Two ion channels, Piezo1 and Piezo2, that are responsive to mechanical forces make up the Piezo channel family. A cation channel called Piezo1 may be opened by mechanical forces such hydrostatic pressure, fluid shear stress, stretching, and other similar phenomena (6–11). Distinct from Piezo2, which is abundant in sensory neurons and other mechanosensitive tissues, Piezo1 could converts mechanical stimuli into signals, thereby regulating vascular tone, trophoblast cell invasion, and adaptive changes in the uterus during pregnancy (12–14). Cryo-EM studies have shown that both channels are composed of a homotrimeric structure with approximately 2500 amino acids arranged in a three-bladed propeller-like configuration, containing 38 transmembrane regions and a central pore, with a sequence homology of 42% (15–17). Mechanical force serves as the endogenous physical activator of Piezo1, whilst its endogenous chemical agonist remains unidentified. Embryo implantation and the start of labor are two of the many pregnancy-related events that include the extensive expression of Piezo1 (18). Other immune cells that express Piezo1 include dendritic cells and macrophages (19).

Piezo1 involves in both the innate and adaptive immune responses. As an intricate and effective system, innate immunity serves as the body’s first barrier against harmful invaders and harm to tissues (20). Piezo1 regulates intracellular Ca²^+^ ion concentrations, which in turn affects immune cell activity and function, making it an important player in innate immunological responses, according to recent studies on immune system processes (21). Piezo1 acts upstream of Ca²^+^ dependent transcriptional programs in myometrial cells (22). An important part of the host defensive system against foreign invaders is adaptive immunity, which is mainly accomplished by activating and regulating B cells and T cells. Researching this mechanism in both normal and abnormal conditions is crucial because to its intricacy and variety. Both B and T cells express Piezo1 channel, which allows them to detect mechanical impulses outside of the cell and translate them into biochemical signals inside the cell that control how the immune system works (23, 24).

Flow shear stress, tissue tension, and other mechanical stimuli are detected by Piezo1 during pregnancy (25). By controlling Ca²^+^ influx, it initiates signaling pathways that play a role in vascular remodeling, uterine contractions, placental function, and fetal development (26). Specifically, in placental trophoblast cells, Piezo1-mediated Ca²^+^ signaling regulates cellular invasion, differentiation, and vascular remodeling capabilities, which are crucial for the proper formation of the placenta and the adaptation of blood flow at the maternal-fetal interface. Research has shown that mechanical stimuli may trigger the release of Ca²^+^ into endometrial epithelial cells via the Piezo1 protein. This mechanism is essential for the implantation of embryos in the early stages of pregnancy (27).At the same time, Piezo1 also regulates uterine contractions and placental blood flow, both of which are essential for ensuring proper fetal growth and development (25). Therefore, exploring the molecular mechanisms through which Piezo1 functions during pregnancy not only helps elucidate its specific roles in the physiological regulation of pregnancy but also provides valuable insights into the mechanisms underlying pregnancy complications. Furthermore, Piezo1 can become a potential target for early intervention and treatment of related pregnancy complications in clinical practice.

In summary, Piezo1’s importance in immunological responses and pregnancy processes is shown by its various roles. New information on the prevention and management of inflammatory disorders and pregnancy difficulties may emerge from further study into Piezo1. Physiological pregnancy processes and pregnancy-related complications are covered in this review, along with the role of immune cells, the regulatory networks for lymphocyte activation in adaptive immunity, the pattern recognition regulatory mechanisms of Piezo1 in innate immune responses, and its expression patterns. Our hope is that this work will lead to fresh ideas for studying Piezo1 during pregnancy and identify potential targets for the early detection and treatment of problems that might arise during this time.

## 2 Structure and function of Piezo1

The unique structure of Piezo1 protein provides the molecular basis for its function as a mechanically gated ion channel. There is no known class of ion channels that have sequence homology with mammalian Piezo1 proteins (15). Research into its mutagenicity, biochemical properties, and structure-function relationships is highly challenging precisely because of these characteristics. Following the discovery that this protein forms homomeric oligomeric complexes, the full-length mouse Piezo1 protein—spanning 2,547 amino acids—became the first Piezo protein to be isolated (28). Crucially, the well-known pore blocker ruthenium red suppresses single-channel activity induced by the recombinant pure Piezo1 protein in asymmetric lipid bilayer membranes (28). These results indicate that the Piezo1 protein itself possesses genuine pore-forming capability (28). Researchers solved a number of Piezo1 structures using cryo-electron microscopy, enabling structure-guided characterizations of their structure–function relationships (29). This is attributable to technological advances in the field of structural biology, which have made it possible to determine the structures of protein complexes, including membrane proteins.

### 2.1 Activation of Piezo1

**Figure 1:**
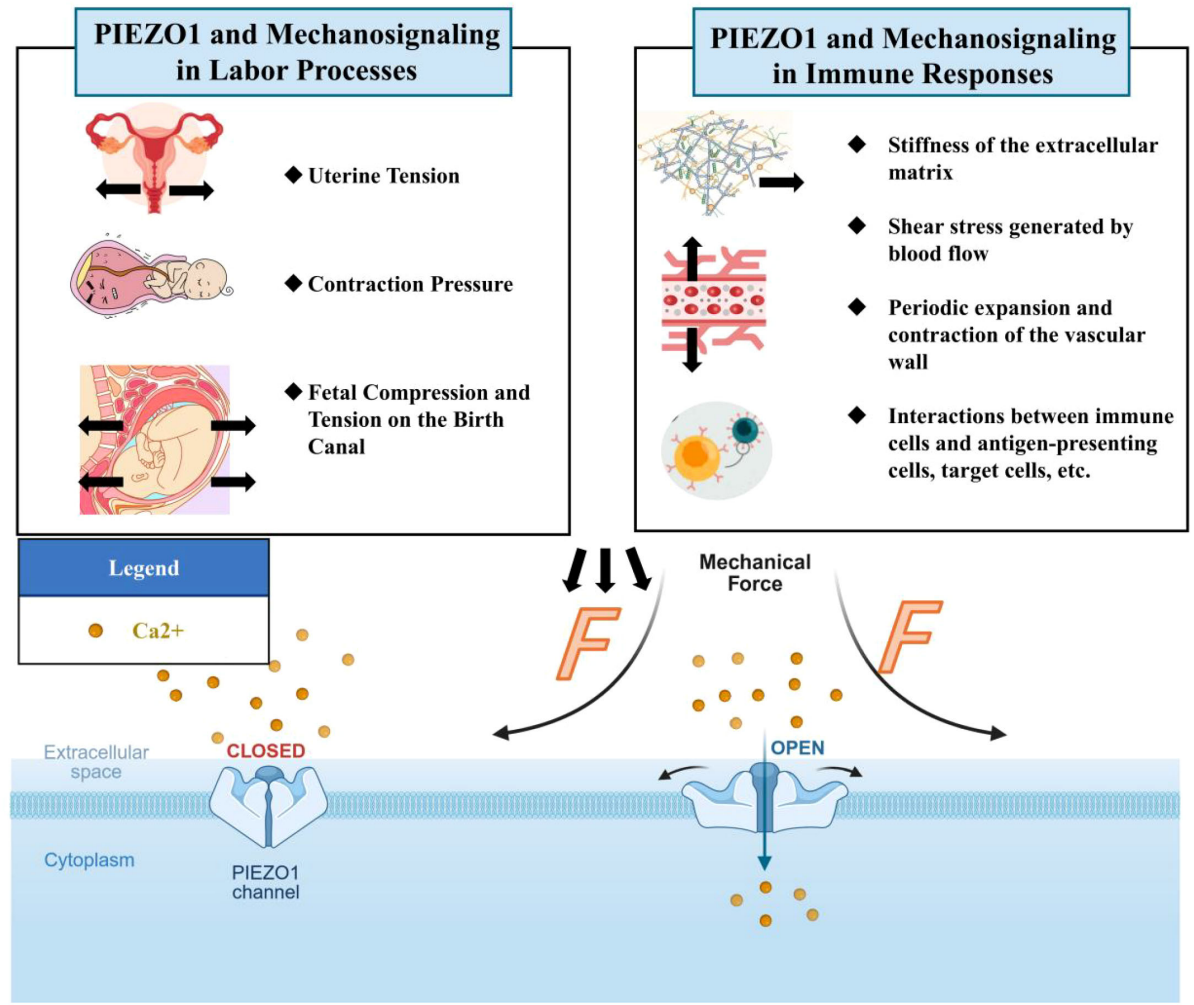
Schematic diagram of Piezo1 channel activation by mechanical force. The Piezo1 channel is a trimeric structure located in the plasma membrane. The mechanical force exerted on the cell membrane causes the opening of the Piezo1 channel, leading to the influx of extracellular Ca2+. (Created in https://BioRender.com).

The mammalian Piezo1 channel generates a mechanically triggered non-selective cation channel with a single-channel conductance of approximately 30 pS and exhibits a slight preference for calcium ions over monovalent sodium ions (15, 28). As shown in **Figure 1**, the role of Piezo1 in immune cells and pregnancy is typically activated by multiple mechanical stimuli. These include externally applied punctures, tensile and shear stresses, locally generated membrane tension, and actin-mediated traction forces originating from within the cell (15, 30, 31). The half-maximal activation concentration of ligand-gated ion channels corresponds to the lateral membrane tension (λ50) required for Piezo1 ion channels to reach half-maximal function, approximately 30 mmHg (15) or 1.4-4.5 pN nm^-1^ (mN m^-1^), respectively (32, 33). When activated in an asymmetric lipid bilayer, isolated Piezo1 channels exhibit an intrinsic sensitivity to membrane tension, a property absent in symmetric lipid bilayers, with an estimated λ50 value of approximately 3.4 pN nm^-^¹ (10).

Wang et al. screened compounds capable of inducing Ca²^+^ responses in Piezo1 or Piezo2-transfected cells, subsequently excluding non-specific compounds that elicited Ca²^+^ responses in all three cell lines (Piezo1, Piezo2, and vector-transfected cells). Ultimately, they identified Jedi1 and Jedi2 as chemical activators of Piezo1, but did not discover compounds that specifically activate Piezo2 (34). Similarly, a study screened approximately 3.25 million compounds using cell-based fluorescence assays, identifying a synthetic small molecule named Yoda1 that acts as an agonist for both human and mouse Piezo1 channels (35). Yoda2 is a more potent Yoda1 counterpart that has been created and synthesized (36). Through separate methods of action, Yoda1 and Jedi1/2 can both directly bind to Piezo1, sensitizing its mechanosensitivity and delaying its inactivation (34). In studies investigating endogenous Piezo1 expression and function, Yoda1 has been widely employed as a surrogate tool for direct patch-clamp recordings of mechanically induced currents. Through molecular docking and mutagenesis studies, a potential Yoda1 binding site was identified within the minimal region of mouse Piezo1 (comprising residues 1961–2062) (37–39). Research indicates that Jedi1/2 activates Piezo1 by acting on key mechanical transduction components within the lever-like apparatus (34). However, structural analysis remains necessary to determine the precise binding sites and activation pathways of Yoda1/2 and Jedi1/2 on Piezo1.

### 2.2 Inactivation of Piezo1

Multiple pharmacological, clinical, and physiological variables influence the inactivation kinetics of Piezo1. Its inactivation rate slows with membrane depolarization, indicating voltage-dependent inactivation dynamics (15). Recent studies indicate that the positively charged lysine residue Lys2479 within the inner helix structure of the mouse Piezo1 ion channel plays a crucial role in this voltage-dependent inactivation process (40). Compared to currents observed in HEK293T cells expressing Piezo1 heterologously, currents mediated by endogenous Piezo1 in multiple cell types—such as endothelial cells, stem cells, renal epithelial cells, and astrocytes—exhibit slower deactivation kinetics (30, 41–43). The upstream Ca^2+^ signaling pathways may be activated if this cell type-specific regulation allowed for an increase in Ca²^+^ influx. This cell type-specific difference in inactivation kinetics suggests the potential existence of specific endogenous regulatory molecules. In fact, beyond voltage and cellular background, certain lipid molecules have also been found to modulate inactivation, such as ceramides. Although the precise mechanism by which ceramides influence Piezo1 inactivation remains unclear, one theory proposes that endogenous sphingomyelin phosphodiesterase 3 (SMPD3) promotes ceramide production in endothelial cells, thereby delaying the inactivation process of Piezo1 (44). In addition, research has shown that proteins phosphorylated by protein kinases A and C (PKA and PKC, respectively) (45) and E-cadherin (46) and the MyoD (myoblast determination) family inhibitor proteins MDFIC and MDFI (46) can post-translationally modify Piezo1/2, delaying its inactivation. For example, Piezo1-related dehydration-associated hereditary macrocytic anemia (DHS) and other human hereditary diseases are partially caused by mutations that slow inactivation kinetics, thereby producing gain-of-function (GOF) effects (47).Additionally, the inactivation kinetics of Piezo1 are also markedly slowed by pharmacological activators, such as Yoda1, Jedi1, and Jedi2 (34, 35). Therefore, to clarify how Piezo1 is diversely regulated *in vivo*, a thorough understanding of its many inactivation mechanisms is necessary.

### 2.3 Structural architecture and domain organization

The functional characterizations, structure-guided mutagenesis, and structural determination have all been carried out using Piezo1 as the model. In 2015, medium-resolution cryo-electron microscopy discovered the propeller-like structure of mouse Piezo1, which is homotrimeric and has three blades. Additionally, its modular construction was shown (48). It consists of a core pore module and three peripheral blades, with 14 resolved transmembranes in each blade, which might function as mechanosensing domains. The C-terminal region forms the true ion-conducting pore with two transmembranes, as previously shown (49). Based on the results of these structural studies, a new approach to structure-guided functional characterizations using mutagenesis and electrophysiology may be considered, and the Piezo1 family of mechanically activated cation channels can be officially recognized. Three different studies of the Piezo1 structure at a resolution of about 4 Å were able to resolve 26 of the 38 transmembranes of each protomer, while the first 12 transmembranes produced by the amino-terminal 500 residues were too flexible to be resolved (17, 50, 51).

## 3 Piezo1 in innate and adaptive immunity

### 3.1 The role of Piezo1 in the innate immune system

In the innate immune system, Piezo1 serves as a key mechanosensor extensively involved in regulating the functions of various immune cells (macrophages, neutrophils, T cells, and so on) (24). In macrophages, Piezo1 detects the physical stiffness of the extracellular matrix or fluid shear stress. Its activation triggers calcium influx that modulates cell polarization and migration, influencing the transition between pro-inflammatory (M1) and anti-inflammatory (M2) phenotypes, thereby playing a central role in inflammation and tissue repair (52). For neutrophils, Piezo1-mediated mechanical sensing is essential for their migration through the vascular endothelium and recruitment to sites of inflammation. Additionally, Piezo1 participates in dendritic cell maturation and antigen presentation processes (53). Specifically, Piezo1 activates downstream signaling pathways, thereby regulating cytokine expression and release, such IL-1β and TNF-α and promotes the polarization of the pro-inflammatory (M1) phenotype in macrophages by activating the YAP/TAZ signaling axis (54). Macrophages on moderately stiff or soft matrices, or those with Piezo1 inhibition, preferentially display the anti-inflammatory (M2) phenotype, in contrast (54–56). Mechanical stretching suppresses the inflammatory response induced by IFNγ/LPS by downregulating Piezo1 expression and upregulating integrin CD11b, but it can enhance IL-4/IL-13-mediated tissue repair (57). Additionally, Piezo1 can form a complex with TLR4, enhancing phagocytosis and mitochondrial ROS production via the CaMKII-Mst1/2-Rac pathway to help clear pathogens (21). Under pathological conditions, oxLDL in atherosclerotic plaques activates Piezo1, promoting macrophage lipid uptake and foam cell formation. In the absence of Piezo1, the MAPK/NF-κB pathway is inhibited, and the Nrf2/HO-1 pathway is activated, thereby alleviating inflammation and plaque progression (58–60). Piezo1 mutations can also cause abnormal macrophage phagocytosis of red blood cells in patients with hereditary polycythemia, leading to iron overload and liver damage (61). During tissue repair, Piezo1 regulates CTSS secretion and Notch signaling to promote liver/kidney fibrosis, while fibroblast activation depends on physical contact between macrophage αvβ3 integrin and Piezo1, triggering the Ca²^+^-NFAT/YAP pathway (62–64).

The function of Piezo1 in innate immune cells extends beyond autonomous cellular regulation. Through intricate intercellular communication, it broadly influences processes such as adaptive immunity, tissue repair, and metabolic reprogramming, thereby shaping the overall immune microenvironment. Mechanical loading triggers Piezo1 in macrophages, leading to the secretion of TGF-β1. This, in turn, promotes the osteogenic differentiation of BMSCs and achieves immune-bone linkage, which is necessary for bone regeneration (65–67).In an ischemic hindlimb model, macrophage Piezo1 inhibits FGF2 secretion via the CaMKII/ETS1 pathway, and its absence enhances angiogenesis and improves perfusion recovery (68). Piezo1 drives the proliferation of ILC3s and IL-17A secretion via the PI3K-Akt-mTOR pathway, promoting the progression of colitis (69, 70). After spinal cord injury, microglial Piezo1 regulates GPX4/ACSL4-mediated ferroptosis, affecting inflammation balance and neural repair, while suppressing T cell infiltration (71). Targeted Piezo1 drugs, through regulation of Ca²^+^ signaling and oxidative stress, inhibit ILC2-driven allergic asthma or ILC3-related intestinal inflammation, providing new therapeutic strategies for immune diseases (70, 72). Furthermore, Piezo1’s association with metabolic reprogramming (such as glycolysis and ferroptosis) further extends its role in immune metabolic regulation (73–75).

Piezo1 functions in innate immune cells with significant subgroup specificity. The mechanosensing of bone marrow-derived macrophages (M_BM) primarily depends on Piezo1-mediated Ca²^+^ influx, while tissue-resident macrophages (M_TR) respond to mechanical stimulation via the TRPV4 channel. This suggests differential regulatory mechanisms of mechanical signals in macrophage subgroups across different microenvironments (76, 77). Neutrophils, as early responders in the innate immune system, also have their migratory behavior regulated by Piezo1. Under the influence of inflammatory chemokines (such as CXCL1), Piezo1 enhances cytoskeletal remodeling through activation of the RhoA/ROCK pathway, promoting directed migration of neutrophils to injury sites (78).

Mechanical stimulation and pharmacological regulation together form a bidirectional intervention system. Mechanical stimulation methods such as low-intensity pulsed ultrasound (LIPUS) and sonodynamic therapy (SDT) can activate Piezo1-mediated Ca²^+^ influx, thereby regulating macrophage ROS metabolism and NF-κB signaling, which leads to the inhibition of inflammation or reprogramming of tumor-associated macrophages (TAMs) toward M1 polarization, enhancing anti-tumor immunity (79–82). In terms of pharmacological regulation, dexamethasone activates SGK1 to enhance store-operated Ca²^+^ entry (SOCE), amplifying Piezo1-mediated Ca²^+^ oscillations and inhibiting the TLR4/NF-κB pathway to alleviate inflammation. However, excessive activation of dexamethasone can lead to mitochondrial damage and macrophage apoptosis (83). Additionally, the peptide inhibitor GsMTx4 can block Piezo1 activity, inhibiting M1 polarization and inflammatory cytokine secretion in macrophages, while the agonist Yoda1 mimics mechanical signals to activate Ca²^+^ signaling, providing a dual-target strategy for immune modulation (84, 85).

Particularly within the vascular and circulatory systems, Piezo1 plays a pivotal role in key processes such as inflammatory exudation, vascular regeneration, and thrombosis by coordinating the mechanical signaling dialogue between endothelial cells and various types of leukocytes. In the context of vascular barrier function, Piezo1 senses leukocyte-induced ICAM-1 clustering and blood flow shear stress, thereby activating Ca²^+^ signaling and SRC/PYK2 kinase phosphorylation, which drives endothelial barrier opening and facilitates leukocyte extravasation (86). When monocytes are stimulated by high shear stress, Piezo1-mediated Ca²^+^ influx activates the integrin Mac-1 and releases more inflammatory cytokines, further exacerbating aortic valve stenosis (87). During vascular regeneration, Piezo1-expressing CD11b+ monocytes can sense the mechanical properties of fibrinogen hydrogels, promoting stable blood vessel branching (88). The function of neutrophils is regulated by Piezo1, with its mediated Ca²^+^ signaling enhancing bacterial clearance through activation of calpain and NADPH oxidase 4 (89), while also regulating shear stress-dependent NETosis, contributing to inflammation in atherosclerosis and bile duct injury following liver transplantation (53, 90, 91). Moreover, Piezo1 regulates macrophage responses to fluid pressure, driving the EDN1-HIF1α signaling axis to promote the clearance of lung infections (92), while NK cells use Piezo1 to sense the stiffness differences of tumor cells, enhancing their killing efficiency (93). In the lymphoid tissue microenvironment, Piezo1 senses the fluid flow in Peyer’s patches (PP), maintaining matrix integrity and ensuring lymphocyte homing and mucosal immune activation (94). Notably, lung inflammation and damage are worsened in acute respiratory distress syndrome (ARDS) when Piezo1 expression is downregulated (95), indicating that Piezo1 might be an important regulatory node in the mechanical balance of innate immunity. These discoveries provide theoretical support for targeting mechanical signaling in the therapy of inflammatory illnesses, since they demonstrate the important function of Piezo1 in innate immune cell migration, inflammation activation, and tissue healing.

Piezo1 functions extend beyond classical immune cells to encompass the hematopoietic system and tissue-specific immune microenvironments, regulating thrombopoiesis and influencing tumor and mucosal immunity. The activation of Piezo1 has been shown in studies to reduce platelet count by preventing megakaryocyte development and polyploidization. Overexpression of Piezo1 is associated with abnormal megakaryocyte phenotypes in primary myelofibrosis (PMF), suggesting that Piezo1 may regulate the homeostasis of the hematopoietic microenvironment through mechanical signals (96). During skin carcinogenesis, Piezo1 senses mechanical changes in the lymphatic microenvironment induced by ultraviolet radiation and dynamically regulates lymphatic vessel density and CD8+ T cell infiltration, affecting immune evasion and progression of cutaneous squamous cell carcinoma (cSCC) (97). In intestinal inflammation, Piezo1 maintains mucosal barrier integrity and regulates immune cell migration, inhibiting the systemic spread of pathogen-induced inflammation (98). Furthermore, according to current literature reviews, macrophages and dendritic cells can sense substrate stiffness and mechanical forces through Piezo1, regulating the release of inflammatory factors (such as IL-12, TGFβ1) and metabolic reprogramming, thereby influencing the cross-regulation between innate and adaptive immunity (99, 100). These findings further expand the biological significance of Piezo1 as a mechanical-immune hub in innate immunity.

Piezo1 also plays a crucial role in neuroimmunology, directly influencing the progression of neuroinflammation and degenerative diseases by regulating microglial function. Studies have reported that microglia can sense the mechanical properties of β-amyloid (Aβ) through Piezo1, enhancing their phagocytic capacity to clear Aβ deposits, thereby delaying the pathological progression of Alzheimer’s disease (AD) (101, 102). In addition, Piezo1 regulates Ca²^+^ signaling to inhibit lipopolysaccharide (LPS)-induced activation of the NF-κB inflammatory pathway, reducing the production of pro-inflammatory factors such as TNF-α and IL-6, thereby alleviating neuroinflammation (103). Under high glucose stress conditions, inhibition of Piezo1 can reduce microglial apoptosis through the JNK1/mTOR signaling pathway, maintaining their homeostasis (104). Piezo1 also regulates microglial migration, controlling their recruitment to damaged sites and thus modulating neuroinflammatory responses (105). In an epilepsy model, Piezo1 is dynamically expressed in the neurovascular unit, and its expression level may correlate with the extent of neuroinflammation, suggesting its involvement in the functional regulation of the blood-brain barrier (106). These findings suggest that Piezo1 is a key molecule in mediating innate immune responses in microglia and can integrate mechanical signals into immune responses (107).

As a core mechanosensitive molecule, Piezo1 senses mechanical signals from the tissue microenvironment (such as shear stress, matrix stiffness, etc.) and participates in various biological processes through complex dynamic interactions with innate immune cells (108). These processes include macrophage polarization, neutrophil migration, endothelial barrier remodeling, and pathogen clearance (21). Piezo1 also plays a role in the development of diseases such as atherosclerosis, sepsis, and fibrosis. Targeting Piezo1 activity can reprogram immune cell phenotypes, inhibit excessive inflammation, and promote tissue repair (109). Its functions in neuroimmunity and the tumor microenvironment further highlight its potential for cross-organ regulation (110). This will lay a theoretical foundation for the development of mechanical signal-mediated immunotherapeutic strategies.

### 3.2 The role of Piezo1 in adaptive immune responses

Piezo1 regulates the activation, differentiation, and effector functions of adaptive immune cells through mechanotransduction. In T cells, Piezo1 functions as a mechanical sensor of the immunological synapse. By sensing the mechanical force generated during TCR-pMHC interactions, it mediates Ca²^+^ influx and activates calpain, promoting cortical actin remodeling and the polarized distribution of integrin LFA-1, thereby optimizing TCR signaling and migration capabilities (111–113). Fluid shear stress (FSS) enhances ZAP70 phosphorylation and NFAT/AP-1 signaling via activation of T cell Piezo1, leading to increased secretion of cytokines such as IL-2 and TNF-α. This suggests that the mechanical microenvironment in the circulatory system may amplify T cell inflammatory responses (114). Notably, the regulation of T cell activation by Piezo1 is dependent on the mechanical strength. A mechanical stress of 50 mmHg can induce NFAT1 nuclear translocation and upregulate CD69 expression in resting CD8+ T cells, but in activated T cells (stimulated with LPS or PMA/ionomycin), the same stress suppresses CD69 expression. This biphasic regulatory property may explain immune disorders in diseases involving abnormal mechanical stress, such as hypertension (115). In addition, Piezo1 has a bidirectional regulatory effect on T cell subsets: its deletion leads to a reduction in CD4^+^ effector memory T cells, inhibiting the progression of experimental autoimmune encephalomyelitis (EAE) and graft-versus-host disease (GVHD) (116), but Piezo1 simultaneously limits Treg expansion by inhibiting TGFβ signaling, thus exacerbating autoimmune pathology (117). In tumor immunity, T cell-specific knockout of Piezo1 leads to a functional defect in CD4^+^ helper T cells and weakens CD8^+^ T cell activation (CD25loPD-1hi phenotype), significantly accelerating tumor progression and reducing the response to immunotherapy. This suggests that Piezo1 is an important factor for T cell-mediated antitumor immunity (118). Piezo1 can also downregulate the traction force of CD8^+^ T cells through the GRHL3-RNF114 axis, inhibiting their cytotoxic function; its antagonist can enhance CAR-T cell infiltration and delay tumor growth (119). Additionally, Piezo1 cooperates with TCR signaling to induce the expression of the transcription factor Osr2, accelerating CD8^+^ T cell terminal exhaustion via epigenetic reprogramming, indicating its role as a “checkpoint” in the tumor mechanical microenvironment (120).

Beyond T cells, Piezo1 also plays a pivotal role in B cell activation and the functional regulation of antigen-presenting cells (such as dendritic cells), collectively shaping the entire course of the adaptive immune response. B cells also rely on Piezo1-mediated Ca²^+^ signaling in response to membrane antigens. By sensing contact-induced changes in membrane tension, Piezo1 promotes cell spreading and antigen presentation, providing a new target for vaccine design (121). In dendritic cells (DCs), Piezo1 integrates the SIRT1-HIF1α metabolic pathway with Ca²^+^ calmodulin phosphatase-NFAT signaling to regulate the IL-12/TGFβ1 secretion balance, driving TH1/Treg cell differentiation and influencing antitumor immune responses (122). Furthermore, antigen-loaded DC cell “backpacks” trigger cytoskeletal remodeling and type I interferon secretion via Piezo1, enhancing T cell proliferation and synergizing with radiotherapy to suppress tumors, thus offering a new paradigm for combined mechanical-immunity-based therapies (123). In therapeutic strategies, ultrasound activation of Piezo1 can remotely regulate CAR-T cell gene expression (124), while targeting Piezo1 to regulate T cell traction forces can enhance cytotoxicity, making it a potential strategy to optimize CAR-T or anti-PD-1 therapies (125). Disease-related studies show that abnormal Piezo1 function is associated with poor prognosis in T cell lymphoma patients (126), while the red blood cell E756del mutation confers malaria resistance by dual regulation of T cell function (127). These findings highlight the pivotal role of Piezo1 in adaptive immune mechanoregulation and open new avenues for targeting mechanical signaling to reshape immune responses.

**Figure 2:**
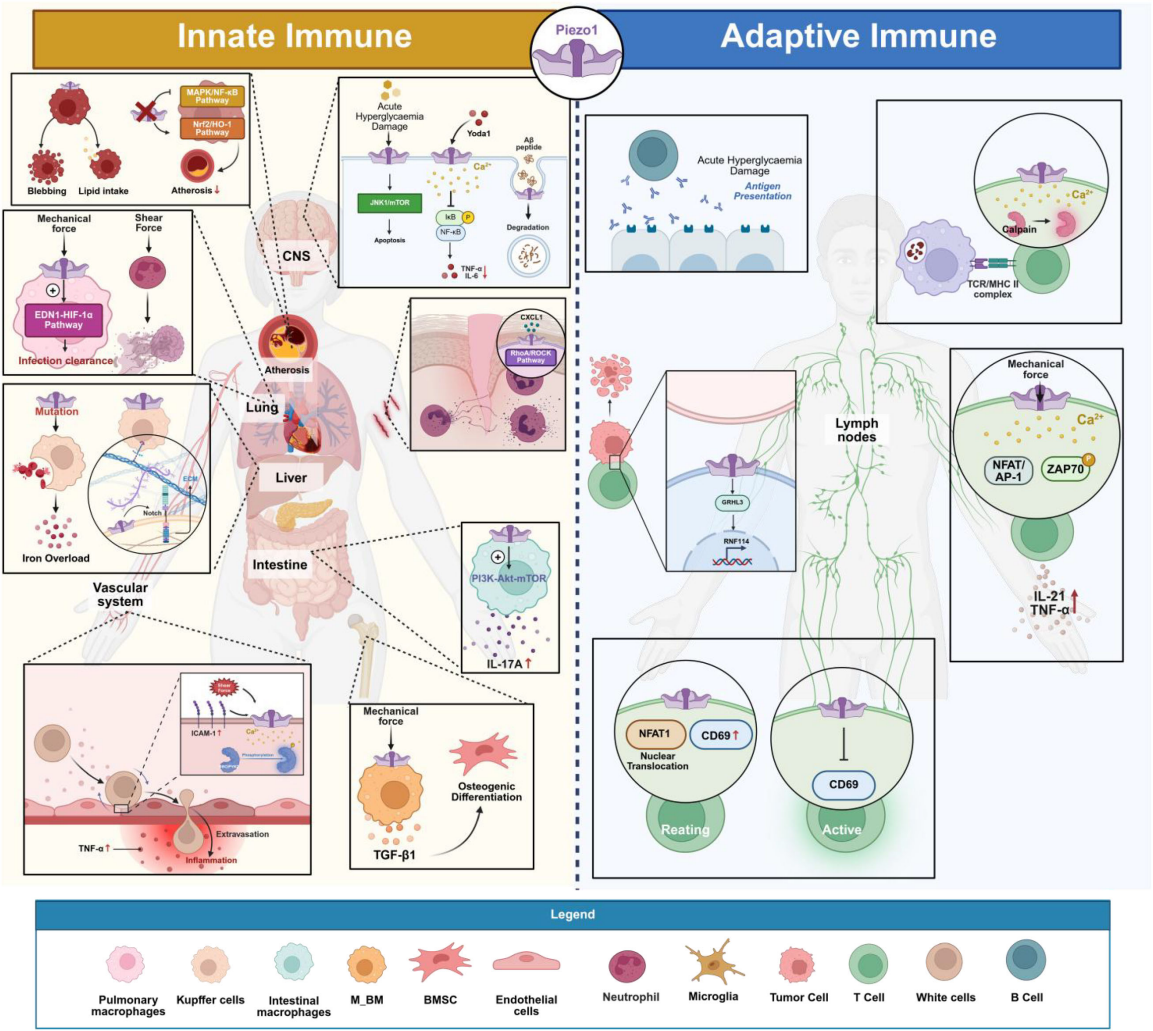
Piezo1 is involved in innate and adaptive immunity. Piezo1 plays a crucial role in processes such as the inflammatory response and pathogen recognition and clearance in innate immunity, as well as the activation, differentiation of lymphocytes and the formation of immunological memory in adaptive immunity. (Created in https://BioRender.com).

In summary, Piezo1 precisely regulates adaptive immune responses through a complex network, and its potential as a therapeutic target is gradually transitioning from basic research to clinical applications. **Table 1** and **Figure 2** showed a novel regulatory layer of Piezo1 in the immune system, deepening our understanding of immune response mechanisms. Future research will need to focus not only on exploring its basic biological functions but also on its clinical application potential, pushing scientific discoveries into practical therapeutic solutions. This is aimed at providing new ideas and methods for improving the treatment outcomes of immune-related diseases.

**Table 1:** Regulatory mechanisms and functions of Piezo1 in the immune system.

Immune system	Cell type	Key functions	Mechanisms/pathways	Disease implications	References
Innate Immunity	Macrophages	- Promotes M1 polarization (IL-1β, TNF-α) via YAP/TAZ. - Inhibits M2 on soft matrices. - Enhances phagocytosis via TLR4-Piezo1-CaMKII-Mst1/2-Rac.	YAP/TAZ, NF-κB, Nrf2/HO-1, Ca²^+^-NFAT/YAP.	Atherosclerosis (foam cell formation), fibrosis, sepsis.	(54–64)
	Neutrophils	- Enhances migration via RhoA/ROCK. - Regulates NETosis under shear stress. - Boosts bacterial clearance via calpain/NOX4.	RhoA/ROCK, Ca²^+^-calpain.	Atherosclerosis, liver transplant rejection, ARDS.	(53, 78, 89–91)
	Microglia	- Clears Aβ plaques in Alzheimer’s. - Inhibits NF-κB via Ca²^+^. - Regulates migration/JNK1-mTOR apoptosis.	Ca²^+^-NF-κB, JNK1/mTOR.	Neuroinflammation, epilepsy, AD.	(101–107)
	NK/ILCs	- ILC3-driven IL-17A via PI3K-Akt-mTOR (colitis). - Enhances tumor cell killing via stiffness sensing.	PI3K-Akt-mTOR, mechanical sensing.	Colitis, cancer immune evasion.	(69, 70, 93)
	Endothelial Cells	- Senses shear stress; promotes leukocyte extravasation via SRC/PYK2. - Monocyte-driven angiogenesis.	Ca²^+^-SRC/PYK2, integrin Mac-1.	Aortic stenosis, vascular regeneration.	(86–88)
Adaptive Immunity	T Cells	- Optimizes TCR signaling via LFA-1 polarization. - Biphasic regulation of activation (NFAT1/CD69). - Limits Tregs (exacerbates autoimmunity).	Ca²^+^-calpain, NFAT/AP-1, TGFβ inhibition.	EAE, GVHD, tumor immunity (CAR-T enhancement).	(111–120)
	B Cells	- Enhances antigen presentation via membrane tension sensing.	Ca²^+^-dependent spreading.	Vaccine design potential.	(121)
	Dendritic Cells (DCs)	- Balances IL-12/TGFβ1 via SIRT1-HIF1α-NFAT. - “Backpack” strategy boosts T cell proliferation.	Metabolic reprogramming, cytoskeletal remodeling.	Antitumor therapy (combined with radiotherapy).	(122, 123)
Therapeutic Strategies	Pharmacological	- GsMTx4 (inhibitor): Blocks M1 polarization. - Yoda1 (agonist): Mimics mechanical activation. - Dexamethasone: Modulates SOCE.	Ca²^+^ signaling, TLR4/NF-κB.	Inflammatory diseases, cancer immunotherapy.	(83–85)
	Physical	- Ultrasound (LIPUS/SDT): Reprograms TAMs to M1, enhances CAR-T.	Piezo1-Ca²^+^-ROS/NF-κB.	Tumor microenvironment modulation.	(79–82, 128)
Cross-Organ Roles	Hematopoiesis	- Inhibits megakaryocyte maturation (↓platelets). - Linked to PMF.	Mechanical sensing in bone marrow.	PMF, thrombocytopenia.	(96)
	Barrier Integrity	- Maintains gut mucosal barrier; limits systemic inflammation. - Regulates lung endothelial function in ARDS.	Ca²^+^-dependent tight junctions.	Inflammatory bowel disease, ARDS.	(95, 98)

M, Macrophage; IL, Interleukin; TNF, Tumor Necrosis Factor; NETosis, Neutrophil Extracellular Trap Cell Death; ILC, Innate Lymphoid Cell; TCR, T Cell Receptor; LFA, Leukocyte Function-Associated Antigen; NFAT, Nuclear Factor of Activated T cell; TGF, Transforming Growth Factor; SOCE, Store-Operated Ca²^+^ Entry; TRPV, Transient Receptor Potential Vanilloid; LIPUS, Low-Intensity Pulsed Ultrasound; SDT, Sonodynamic Therapy; TAM, Tumor-Associated Macrophage; CAR-T, Chimeric Antigen Receptor T-Cell; PMF, Primary Myelofibrosis; ARDS, Acute Respiratory Distress Syndrome.

## 4 The role of Piezo1 in pregnancy

### 4.1 The role of Piezo1 in uterine arteries and placental blood vessels

During vascular adaptation in pregnancy, Piezo1 serves as a core mechanical force sensor, directly responding to hemodynamic changes and regulating vascular function. The uterine and maternal-fetal interface during pregnancy is in a dynamic mechanical stress microenvironment (4). The continuous expansion of the amniotic cavity directly exerts tension on the uterine wall, while the increase in maternal circulating blood volume and the hemodynamic load caused by vascular dilation together form a complex biomechanical regulatory network (129). Urinary artery blood flow increases, which causes shear stress to rise. Changes in shear stress are detected by endothelial cells (ECs) in the artery wall, which then trigger pathways that lead to vasodilation (130). Afterwards, the uterine blood arteries change structurally and functionally to accommodate the increasing blood flow (130). According to research by John L et al., Piezo1 is expressed at higher levels during pregnancy, and its activation leads to vasodilation via nitric oxide (NO). This protein is located in uterine arteries, mostly in endothelial cells but also in other types of cells (Vascular smooth muscle cells, trophoblast cells, pericytes) (131). Animal studies have confirmed this procedure, according to other researchers (132).

The function of Piezo1 is equally critical in the specialized vascular bed of the placenta, and its dysregulation is closely associated with the pathophysiological mechanisms underlying various gestational vascular complications. The Piezo1 channel is a mechanosensor in human fetal placental endothelial cells (133). It is also seen in uterine arteries. Nitric oxide (NO) is produced by vascular endothelial cells in response to shear stress from blood flow, which dilates placental blood channels and increases blood supply to the fetus. Morley LC discovered that healthy fetal placental endothelial cells (FpECs) respond to increased blood flow by signaling NO; however, FpECs from newborns who are small for gestational age (SGA) had enhanced baseline NO signaling, suggesting a compensatory mechanism (134). Anatomical alterations in the placental blood arteries, endothelial dysfunction, hypoxia, which impacts the FSS balance (135), and placental malfunction resulting to fetal growth restriction (FGR) are all factors in this condition. To further understand how to treat placental insufficiency with FGR, more studies examining the endothelial response to FSS are needed (136). Recent studies show that in individual FpECs, Piezo1 and TRPV4 are co-expressed and jointly mediate NO generation and barrier integrity in placental endothelium. Abnormal co-regulation of these channels is associated with early-onset preeclampsia (EPE) functional damage. Combined channel activation can induce pathological events (such as a significant reduction in barrier integrity), and FpECs from EPE are more sensitive to this. RNA-Seq data suggest that endothelial arachidonic acid (AA) metabolism dysregulation may mediate this damage. Targeting Piezo1 and TRPV4 or balancing placental endothelial AA metabolism could become a novel therapeutic strategy for EPE (137).

A lack of dynamic analysis connecting vascular adaptive changes to disease progression, along with other factors, makes it difficult to understand how Piezo1 functions under pathological conditions, such as fetal growth restriction or preeclampsia, even though recent studies have shown that it plays a significant regulatory role in vascular function during pregnancy. A groundwork for the creation of secure and efficient treatment methods may be laid by further investigation into the shared processes of abnormal pregnancies and the interconnected regulatory network spanning mechanical sensing and vascular remodeling.

### 4.2 The role of Piezo1 in the initiation of labor

The uterine myometrium must maintain a state of quiescence during pregnancy in order to adjust to the progressive changes in uterine tension (138). Piezo1 expression was four times greater in the myometrium of women in labor (TL) and fourteen times lower in women experiencing preterm labor (PTL), according to research by Barnett et al., although levels in the myometrium of non-pregnant women and preterm women at term without labor were comparable. It may be inferred from this that its expression is closely linked to the start of labor (18). Additional research has shown that Piezo1 is the principal channel in the mouse myometrium and primary uterine smooth muscle cells (pUSMCs) that is responsive to mechanical stress; its expression levels rise during gestation. An LPS-induced preterm birth model may be used to test whether blocking or knocking down Piezo1 can extend pregnancy. Yoda1 activation raises pUSMC Ca²^+^ levels, but Gsmtx4 activity lowers these levels and inhibits COX-2 and inflammatory factor expression in pUSMCs treated with LPS. Based on these results, Piezo1 seems to play an important role in regulating uterine function, and increasing its expression may hasten labor by triggering inflammation and myometrial activity (18). To reduce inflammation-related preterm labor, our work highlights the possibility of targeting Piezo1 as a therapeutic method (139).

The exact regulatory mechanisms of Piezo1’s function in uterine contractions and inflammatory responses remain unclear, despite the growing body of scientific data linking Piezo1 to labor induction (22). Preterm birth may be better prevented and treated if future studies thoroughly examine the relationship between mechanical signals and the labor regulatory network.

### 4.3 The impact of Piezo1 mutations on fetal development

The integrity of Piezo1 gene function is crucial for normal fetal development, and its loss-of-function mutations are a major cause of severe developmental abnormalities such as non-immune hydrops fetalis. According to recent studies, GLD is an extremely uncommon form of lymphedema that causes widespread lymphatic swelling, dilatation of the lymphatic vessels in the intestines and lungs, fluid around the heart (pericardial effusion), and pleural effusion. Prenatal non-immune edema is one possible symptom (140). When it comes to non-immune fetal edema, prenatal exome sequencing has shown Piezo1 as the most prevalent monogenic etiology (141). Reduced mechanical sensitivity of tissues and impaired alignment of endothelial cells in response to shear stress are the results of Piezo1 loss-of-function (142). Shear stress activates signaling pathways that enhance the maturity of lymphatic vessels and the creation of valves, demonstrating mechanotransduction as an important regulator in lymphatic development (143). Several reports have linked non-immune fetal edema to Piezo1 mutations (144–148). Whole exome sequencing is a necessary diagnostic tool for identifying the cause of non-immune fetal edema and can be combined with imaging assessments, such as ultrasound, to evaluate prognosis (149–152). Piezo1 gene mutations may also be associated with fetal ascites or the regulation of bone density/strength (153, 154). Additionally, Piezo1 mediates chloride ion regulation in the lumen, affecting lung branching morphogenesis (155).

Although the association between Piezo1 mutations and fetal phenotypes has been confirmed, a significant gap remains in the research pathway from elucidating the mechanism to clinical translation. Research on the association between Piezo1 mutations and fetal developmental abnormalities is still in the phase of phenotypic description. There is a lack of systematic verification of the genotype-phenotype correlation, and the long-term health effects of mutations on the fetus have not been fully tracked. Current prenatal diagnostic technologies are insufficient for accurately assessing the functional consequences of mutations, and targeted intrauterine treatment methods are still lacking. Future studies need to establish a multidimensional research framework that integrates genetics, developmental biology, and clinical data, while also exploring the frontier applications of technologies such as gene editing, in order to provide new approaches for improving mutation-related fetal outcomes.

### 4.4 The role of Piezo1 in other pregnancy processes

The uterine endometrium and the implanted embryo engage in a complicated interplay that is necessary for a successful pregnancy (156). Piezo1 is abundantly expressed in uterine epithelial cells (EEC) of both humans and mice, according to research by Hennes A et al. Both the intracellular Ca²^+^ content and the current density in EEC may be increased by the pharmacological agonist Yoda1 for Piezo1.This suggests that EEC functionally expresses the mechanosensitive Piezo1 channel, which may become a potential target for developing new therapeutic approaches to improve implantation success rates (27). For women with subsequent pregnancies after a cesarean section, uterine scars are associated with placental implantation abnormalities (PAS), characterized by invasive trophoblasts entering the uterus. A repeat cesarean section is often required for subsequent deliveries (157). Researchers constructed a uterine scar model and confirmed that the scar matrix activates mechanosensitive ion channels, including Piezo1, through glycolysis-driven cellular contraction. In order to translocate NF-κB to the nucleus and stabilize MafG, the activation of Piezo1 raises intracellular Ca²^+^ activity and activates protein kinase C. Interleukin-8 (IL-8) and granulocyte colony-stimulating factor (G-CSF) are produced as a consequence of this decidua inflammatory change. These factors chemotactically attract invasive trophoblasts to the scar site, which initiates PAS (157).

### 4.5 The role of Piezo1 in trophoblast cell fusion

**Figure 3:**
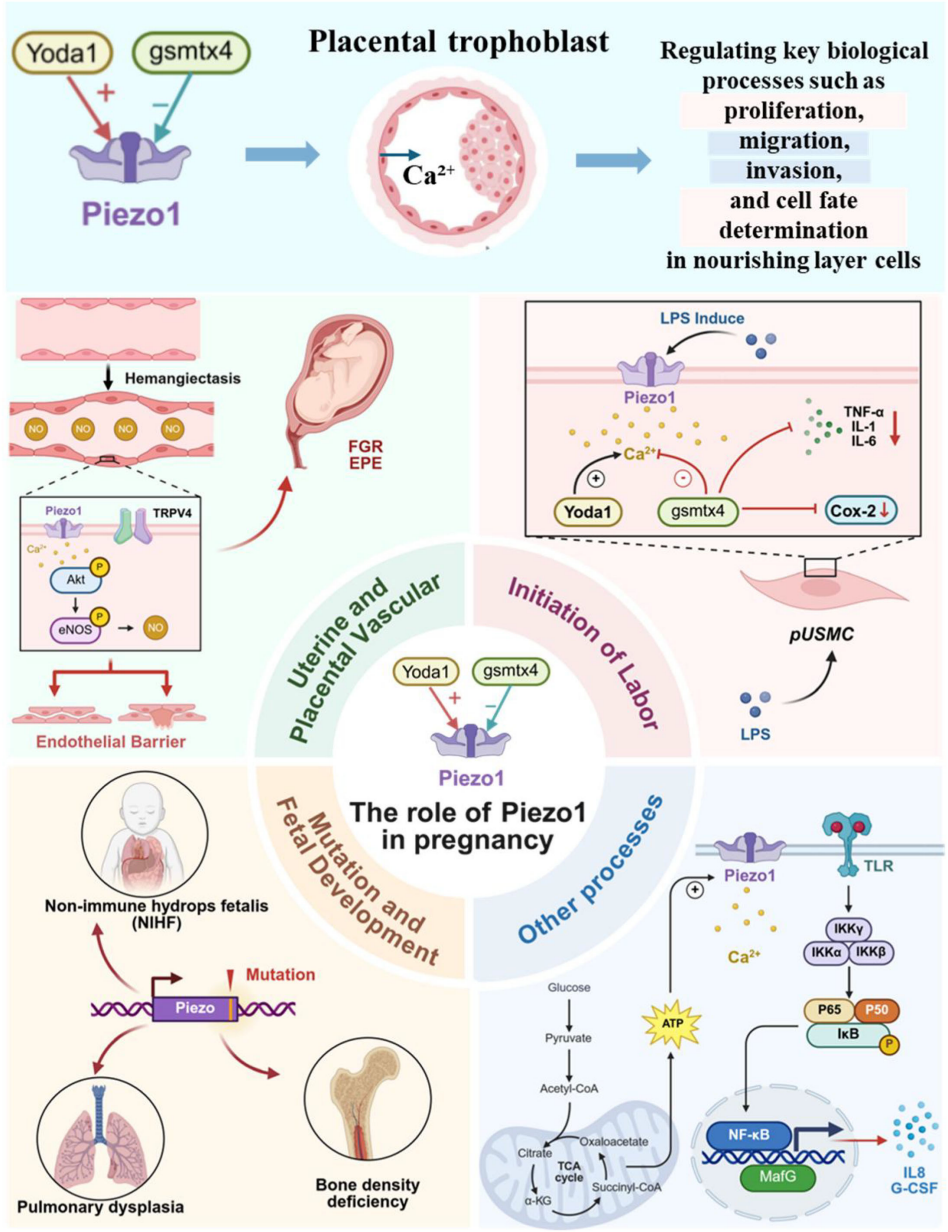
Schematic diagram of the role of Piezo1 in pregnancy. Piezo1 affects important pregnancy processes such as placental trophoblast, embryo implantation, remodeling of the uterine spiral arteries, placental vascular development, and the initiation of labor, etc. (Created in https://BioRender.com).

The involvement of Piezo1 in placental trophoblast cells has recently been the focus of research, shifting attention from its function in vascular endothelial cells, which had been the primary focus of earlier investigations. High embryonic mortality is also seen when Piezo1 is specifically knocked out in trophoblasts, according to the researchers. This mechanism differs from the aforementioned vascular endothelial-related pathways, Piezo1 mediates Ca²^+^ ion influx to activate TMEM16F lipid scramblase, promoting phosphatidylserine externalization and providing a key “fusion signal” for trophoblast fusion, its deletion disrupts the trophoblast fusion process, impairs syncytiotrophoblast formation, and ultimately leads to embryonic lethality (26). **Table 2** listed the regulatory mechanisms and functions of Piezo1 during pregnancy, including the gestational process, key findings, mechanisms/pathways, clinical implications, and relevant references (130–134, 136, 137, 140, 141, 143–153, 158). As shown in **Figure 3**, Piezo1 regulates embryo implantation, placental vascular remodeling, uterine contractions, and fetal development through mechanochemical signal transduction. Its abnormal function is closely related to various pregnancy complications (PAS, FGR, preterm birth, NIHF), offering new molecular targets for therapeutic interventions (such as for preterm birth and early-onset preeclampsia). Future research needs to further elucidate its precise regulatory mechanisms in pathological pregnancies, paving the way for clinical translation applications.

**Table 2:** Regulatory mechanisms and functions of Piezo1 in pregnancy.

Pregnancy process	Key findings	Mechanisms/pathways	Clinical implications	References
Uterine Artery & Placental Vascular Remodeling	- Piezo1 mediates vasodilation via NO in uterine arteries. - Upregulated during pregnancy. - Co-expressed with TRPV4 in placental endothelium.	Shear stress → Piezo1 → NO release. Dysregulated AA metabolism in preeclampsia.	FGR, EPE.	(130–134, 136, 137)
Labor Initiation	- Piezo1 expression ↑ in TL but ↓ in PTL. - Yoda1 (agonist) ↑ Ca²^+^/COX-2; GsMTx4 (inhibitor) ↓ inflammation.	Ca²^+^-dependent myometrial activation. LPS-induced NF-κB/COX-2 signaling.	Therapeutic target for preterm labor.	(18, 139)
Fetal Developmental Defects	- Piezo1 mutations cause NIFH and lymphatic dysplasia. - Impaired endothelial alignment under shear stress.	Loss-of-function → reduced mechanosensitivity → lymphatic/vascular defects.	Prenatal diagnosis via exome sequencing; potential gene therapy targets.	(140, 141, 143–153, 158)
Embryo Implantation & Uterine Scarring	- Piezo1 expressed in uterine EECs; Yoda1 ↑ Ca²^+^ currents. - Uterine scars activate Piezo1 → NF-κB/IL-8 → trophoblast recruitment (PAS).	Glycolysis-driven contraction → Piezo1 → Ca²^+^-PKC-NF-κB-MafG.	PAS; improving implantation success.	(27, 157)
Trophoblast Cell Fusion	-Piezo1 expressed in placental trophoblast cells. - Specific knockout of trophoblastic Piezo1 leads to high embryonic mortality.	Piezo1 activation → Ca²^+^ influx → TMEM16F lipid scramblase activation → phosphatidylserine externalization → trophoblast fusion and syncytiotrophoblast formation.	placental disorders	(26)

NO, Nitric Oxide; FGR, Fetal Growth Restriction; EPE, Early-onset Preeclampsia; TRPV, Transient Receptor Potential Vanilloid; AA, Arachidonic Acid; TL, Term Labor; PTL, Preterm Labor; NIFH, Non-immune Fetal Hydrops; EEC, Epithelial Cell; PAS, Placenta Accreta Spectrum.

## 5 The therapeutic potential and future directions of Piezo1

As a crucial mechanosensitive ion channel, Piezo1 has become a valuable therapeutic target for the management of a number of illnesses. Targeting Piezo1 in various clinical situations has great promise for tissue regeneration, neuroprotection, and cancer treatment in addition to modifying immunological responses (159).

The foundation for realizing this potential lies in the specific pharmacological modulation of Piezo1 channel activity. Piezo1-targeting medications, such as the peptide inhibitor GsMTx4 and the agonist Yoda1, control oxidative stress and Ca²^+^ signaling in inflammatory disorders, providing novel approaches to the treatment of intestinal inflammation linked to ILC3 and ILC2-driven allergic asthma (160, 161). Dexamethasone reduces inflammation by blocking the TLR4/NF-κB pathway and activating SGK1 to improve Piezo1-mediated Ca²^+^ oscillations, albeit overactivation may have negative consequences (58). In autoimmune diseases, Piezo1 inhibition decreases CD4+ effector memory T cells, which improves graft-versus-host disease (GVHD) and experimental autoimmune encephalomyelitis (EAE) (89).

Notably, the regulatory strategy of Piezo1 demonstrates unique and flexible application prospects in tumor immunotherapy. Piezo1 regulation exhibits multifaceted promise in cancer immunotherapy. When CD11b+ dendritic cells lose Piezo1, antigen absorption and MHC class I expression are increased, which encourages CD8+ T cell cross-activation and inhibits tumor growth and metastasis (19). Additionally, targeting Piezo1 promotes tumor infiltration, inhibits T cell exhaustion, increases CAR-T cell cytotoxicity, and slows the growth of tumors (90, 91). Ultrasound-mediated activation of Piezo1 enables remote modulation of CAR-T cell gene expression (96), while antigen-loaded DC “backpacks” promote T cell proliferation via Piezo1 activation and synergize with radiotherapy to suppress tumors, providing new avenues for combined immunotherapies (95).

Beyond the fields of immunology and oncology, Piezo1 also plays a pivotal role in pathological processes within the nervous system, offering a novel perspective for interventions targeting neurodegenerative diseases. The function of Piezo1 in neurodegenerative disorders is noteworthy. By using Piezo1 to sense the mechanical characteristics of amyloid-β (Aβ), microglia improve phagocytosis and provide a new avenue for treating Alzheimer’s disease (AD) (77, 78). Targeted delivery of GsMTx4 using CAQKERM@GsMTx4 nanoparticles alters microglial polarization, attenuates neuroinflammation and neuronal damage, and has intriguing therapeutic potential in a subarachnoid hemorrhage (SAH) model (162).

The impact of Piezo1 in the field of tissue engineering and regenerative medicine is equally complex and profound. The effects of Piezo1 intervention in tissue regeneration and repair are varied. While GsMTx4-loaded GelMA hydrogel suppresses Piezo1 and the Apelin signaling pathway, promoting tendon regeneration and increasing biomechanical characteristics, Piezo1 overexpression after tendon injury encourages heterotopic ossification (163). Loss of Piezo1 in macrophages improves angiogenesis and perfusion recovery in a model of hindlimb ischemia, indicating that it may be a pro-angiogenic target (164). Furthermore, intravitreal injection of GsMTx4 delays axial elongation and improves refractive status in myopia development by inhibiting ferroptosis and Piezo1 overexpression (165).

Piezo1 exhibits a wide range of therapeutic possibilities in the obstetrics domain. First, by controlling the channel or its downstream metabolic pathways (like arachidonic acid metabolism) to improve placental vascular dysfunction, targeted modulation of Piezo1 channel activity may provide new treatment approaches for preterm birth and early-onset preeclampsia (101, 106). Second, a new target for intervention for fetal growth limitation is provided by the Piezo1-mediated mechanotransduction process (105). Additionally, a key area of future research will be the genetic diagnosis and intrauterine treatment of fetal hydrops caused by Piezo1 mutations (113, 120–123). Through its regulation of trophoblast fusion (26) and endometrial mechanosensation (27), Piezo1 provides new insights for enhancing embryo implantation and preventing aberrant placental adhesion in the setting of embryo implantation and placental development. In order to promote the use of Piezo1-based precision therapeutic approaches in obstetrics, future research should incorporate multi-omics analysis and dynamic monitoring technologies to methodically clarify the entire regulatory network from mechanical sensing to pathological development.

In conclusion, Piezo1 is essential for several disease processes, including as tissue regeneration, inflammation, cancer, autoimmune, and neurological conditions. Its activity modification shows multi-level therapeutic significance and offers crucial guidance for upcoming medication development and treatment strategy enhancement.

## 6 Conclusion

Piezo1, a mechanosensitive ion channel, has generated a lot of buzz as of late, because to the important functions it plays in immune cells and during pregnancy. This review emphasizes that Piezo1 is involved in immunological control and the process of pregnancy, rather than just being a passive sensor of mechanical stimulation. While Piezo1 controls the mechanical force-sensing threshold of T cells and takes role in B-cell activation and antibody generation in adaptive immunity, it has both pro-inflammatory and anti-inflammatory actions in innate immunity. Its functional diversity establishes it as a key hub in the mechanosensory-immune interaction network, though its effects are highly dependent on cell type, mechanical microenvironment, and the fine regulation of pathological contexts.

It is noteworthy that Piezo1 also exerts regulatory effects during the specific physiological process of pregnancy. Research indicates that Piezo1 may participate in regulatory processes during early pregnancy. For instance, during the embryonic implantation phase, Piezo1 in uterine epithelial cells potentially influences implantation success rates by modulating Ca²^+^ signaling; in uterine and placental vasculature, it senses hemodynamic changes to mediate NO-dependent vasodilation and vascular remodeling, ensuring adequate placental perfusion—dysregulation of Piezo1 can lead to placental endothelial dysfunction. During labor initiation, dynamic changes in Piezo1 expression in uterine muscle suggest that its overexpression may promote preterm birth by enhancing uterine contractility and inflammation. Additionally, Piezo1 gene mutations are closely linked to non-immune fetal hydrops (NIHF) and lymphatic vessel developmental abnormalities.

Based on the aforementioned functions, Piezo1’s pivotal role in maintaining pregnancy homeostasis and its clinical translational potential are becoming increasingly evident. Piezo1 is an important mechanosensitive channel that affects immune cell activity; immunological tolerance and balance between the mother and fetus are prerequisites for a healthy pregnancy. Its therapeutic target potential is shown by its extensive participation in these processes. The pathogenic mechanisms of Piezo1 in immune-related pregnancy complications like recurrent miscarriage (RM), preeclampsia (PE), and PL should be the focus of future research into its role in pregnancy immune regulation. This will hopefully lead to the development of new approaches for preventing and treating these conditions through immune modulation.
